# Transcriptome Sequencing Reveals Key Pathways and Genes Associated with Cisplatin Resistance in Lung Adenocarcinoma A549 Cells

**DOI:** 10.1371/journal.pone.0170609

**Published:** 2017-01-23

**Authors:** Yani Fang, Cheng Zhang, Tong Wu, Qi Wang, Jinhui Liu, Penggao Dai

**Affiliations:** 1 National Engineering Research Center for Miniaturized Detection Systems, College of Life Science, Northwest University, Xi’an, PR China; 2 Shaanxi Lifegen Co. Ltd., Xi’an, PR China; University of South Alabama Mitchell Cancer Institute, UNITED STATES

## Abstract

Acquired resistance to cisplatin-based chemotherapy frequently occurs in patients with non-small cell lung cancer, and the underlying molecular mechanisms are not well understood. The aim of this study was to investigate whether a distinct gene expression pattern is associated with acquired resistance to cisplatin in human lung adenocarcinoma. Whole-transcriptome sequencing was performed to compare the genome-wide gene expression patterns of the human lung adenocarcinoma A549 cisplatin-resistant cell line A549/DDP with those of its progenitor cell line A549. A total of 1214 differentially expressed genes (DEGs) were identified, 656 of which were upregulated and 558 were downregulated. Functional annotation of the DEGs in the Kyoto Encyclopedia of Genes and Genomes database revealed that most of the identified genes were enriched in the PI3K/AKT, mitogen-activated protein kinase, actin cytoskeleton regulation, and focal adhesion pathways in A549/DDP cells. These results support previous studies demonstrating that the pathways regulating cell proliferation and invasion confer resistance to chemotherapy. Furthermore, the results proved that cell adhesion and cytoskeleton regulation is associated with cisplatin resistance in human lung cancer. Our study provides new promising biomarkers for lung cancer prognosis and potential therapeutic targets for lung cancer treatment.

## Introduction

Lung cancer is the most prevalent malignancy worldwide, accounting for the highest incidence and mortality rates of all cancer types[1]. In the clinical treatment of lung cancer, chemotherapy can be used as adjuvant therapy either alone or in combination with radiation[2]. For decades, cisplatin has been used as the first-line drug for chemotherapeutic administration in cases of advanced and metastatic lung cancer[3]. However, the prognosis for patients with advanced lung cancer remains poor, with a median survival time of 8–11 months, a 1-year survival rate of 30–45%, and a 5-year survival rate of <5%[4]. Acquired resistance after prolonged exposure to cisplatin is considered as one of the primary reasons for the failure of chemotherapy[5].

Nevertheless, the underlying mechanisms of acquired resistance to cisplatin are not well understood. The suggested mechanisms reported to date can be divided into four main categories: decreased drug absorption as well as increased drug loss, increased DNA repair, inactivated apoptotic pathways, or activated pathways that are not directly engaged by cisplatin, but rather compensate for the cisplatin toxicity and help the cell escape[6]. Therefore, a more comprehensive understanding of cisplatin resistance and related targeted therapies are urgently needed to improve the clinical treatment of lung cancer patients.

With the rapid development of sequencing technology, next-generation sequencing (NGS) platforms exhibiting greater speed and higher throughput at lower costs have gradually replaced the traditional technologies. NGS facilitates the deep sequencing of whole cancer genomes for the discovery of novel therapeutic biomarkers, helping to consequently build a solid foundation for comprehensive studies of cancer pharmacogenetics. Furthermore, apart from DNA sequencing, NGS allows for detailed analyses of the whole epigenome and transcriptome, thus profoundly revealing the multilevel regulation networks of the human genome[7]. Remarkably, gene expression profiles as well as detection of mutations, sequence aberrations, alternative splice variants, and RNA editing events revealed by transcriptome sequences have provided valuable resources for studies investigating therapeutic biomarkers of cancer[8].

Therefore, in the present study, whole-transcriptome sequencing was performed to compare the gene expression profiles between a human lung adenocarcinoma cisplatin-resistant cell line (A549/DDP) with its progenitor (A549), thus revealing potential biomarkers associated with cisplatin resistance in lung cancer.

## Materials and Methods

### Cell culture

Cell lines used in this study were obtained from the Chinese Academy of Sciences Committee on Type Culture Collection Cell Bank (Shanghai, China). The human lung cancer A549 cells and the human hepatoma HepG2 cells were cultured in Roswell Park Memorial Institute medium 1640 (Gibco, Carlsbad, CA, USA) and Dulbecco's modified Eagle's medium (Gibco) respectively, supplemented with 10% (v/v) fetal bovine serum (Gibco), 100 U/mL penicillin (Gibco), and 100 U/mL streptomycin (Gibco) at 37°C in a humidified atmosphere containing 5% CO_2_. The cisplatin-resistant cell lines A549/DDP and HepG2/DDP were established from their parental cell lines by step-dose selection *in vitro*. A549 cells were treated with cisplatin at concentrations ranging from 1μM to 8μM over a period of 4 months, while the concentrations used for HepG2 were ranging from 0.1μg/mL to 2μg/mL. The A549/DDP cells and the HepG2/DDP cells were cultured in medium containing cisplatin to maintain the drug-resistant phenotype (1μM and 0.5μg/mL respectively) and then cultured in drug-free medium for over 2 weeks before subsequent experiments.

### Derivation of single cell-derived clones

Single cell-derived clones were established according to the method described by Bi et al[9]. After enzyme digestion and filtration, A549/DDP and A549 cells were suspended in culture medium. The cells were then seeded in 96-well tissue culture plates (Thermo Scientific, Wilmington, DE, USA) at a density of 2 cells/well (200μl/well). After 24h, the plates were scored under the microscope. Wells containing only one cell were marked for further analysis. The cells were kept in the original culture medium at 37°C with 5% CO_2_. After 2–3 weeks, the single cell-derived clones were harvested and transferred to 24-well and six-well tissue culture plates (Thermo Scientific) for expansion. In total, 20 clones were established for A549/DDP cells and 15 clones were established for A549 cells.

### Cytotoxicity assay

A549 and A549/DDP single cell-derived clones were seeded in a 96-well plate at a density of 5 × 10^4^ cells per well. After incubation for 24 h, the used medium was replaced by fresh medium with cisplatin at concentrations of 0, 1, 2, 4, 6, 8, and 16μg/mL in the presence of 1% fetal bovine serum for another 48 h of incubation. Cell viability was tested using the CCK-8 assay. The absorbance of each well was measured at 450 nm on a microplate reader. The proliferation rate was defined in terms of the percentage of each group of surviving cells compared with the untreated group for both cell lines.

### RNA extraction

Total RNA was isolated from A549 and A549/DDP single cell-derived clones using TRIzol reagent (Invitrogen, Carlsbad, CA, USA). RNA degradation and contamination was monitored on 1% agarose gels. RNA purity was checked using the NanoPhotometer spectrophotometer (Implen, Westlake Village, CA, USA). RNA concentration was measured using Qubit RNA Assay Kit on a Qubit2.0 fluorometer (Life Technologies, Carlsbad, CA, USA), and integrity was assessed using the RNA Nano 6000 Assay Kit of the Agilent Bioanalyzer 2100 system (Agilent Technologies, Santa Clara, CA, USA).

### Transcriptome sequencing

A total of 3 ng RNA per sample was used as input material for the RNA sample preparations. Sequencing libraries were generated using NEB NextUltr RNA Library Prep Kit for Illumina (New England Biolabs, Ipswich, MA, USA) following the manufacturer’s recommendations. In brief, mRNA was purified from total RNA using poly-T oligo-attached magnetic beads. Fragmentation was carried out using divalent cations under elevated temperature in NEBNext First Strand Synthesis Reaction Buffer (5X). First-strand cDNA was synthesized using random hexamer primers and M-MuLV Reverse Transcriptase. Second-strand cDNA synthesis was subsequently performed using DNA Polymerase I and RNase H. Remaining overhangs were converted into blunt ends via exonuclease/polymerase activities. After adenylation of the 3ʹ ends of DNA fragments, NEBNext Adaptor with a hairpin loop structure was ligated to prepare for hybridization. In order to select cDNA fragments of preferentially 150–200 bp in length, the library fragments were purified with the AMPure XP system (Beckman Coulter, Beverly, MA, USA). Then, 3μl of USER Enzyme (New England Biolabs) was used with size-selected, adaptor-ligated cDNA at 37°C for 15 min followed by 5 min at 95°C before polymerase chain reaction (PCR). PCR was performed with Phusion High-Fidelity DNA polymerase, Universal PCR primers, and Index (X) Primer. Finally, PCR products were purified (AMPure XP system) and library quality was assessed on the Agilent Bioanalyzer 2100 system. The clustering of the index-coded samples was performed on a cBot Cluster Generation System using TruSeq PE Cluster Kit v3-cBot-HS (Illumina) according to the manufacturer’s instructions. After cluster generation, the library preparations were sequenced on an Illumina Hiseq 2000 platform and paired-end reads were generated.

### Sequencing data analysis

Gene expression levels were estimated using the RSEM package[10]for each sample. Clean data were mapped back onto the assembled transcriptome. The read count for each gene was obtained from the mapping results. Differential expression analysis of two samples was performed using the DEGseq R package. The *p* value was adjusted using the q value. A q value < 0.005 and |log2 (fold change)| >1 was set as the threshold for significantly differential expression.

### Function annotation of differentially expressed genes (DEGs)

The databases used to annotate the function of identified DEGs included Clusters of Orthologous Groups (COG), Gene Ontology (GO), and Kyoto Encyclopedia of Genes and Genomes (KEGG). The query unigene sequences were then matched with the subject sequences in the multiple databases using BLAST (BLASTX tool for proteins and BLASTN tool for nucleotides) at an E-value cut-off of e-5 (<0.00001). GO enrichment analysis of the DEGs was implemented by the GOseq R packages based on the Wallenius non-central hyper-geometric distribution[11]. After achieving GO annotation for every unigene, WEGO software (http://wego.genomics.org.cn/cgi-bin/wego/index.pl/) was used to perform GO classification and draw a GO tree. The classification of DEGs into the functional pathways was conducted by KEGG analysis. The KEGG automatic annotation server was used for KEGG Orthology (KO) and KEGG pathway annotation. Similarly, a BLASTX search against the COG database resulted in the classification of unigenes into COG functional groups[12, 13]. KOBAS[14]software was used to test the statistical enrichment of DEGs in the KEGG pathways.

### Quantitative reverse transcription (qRT)-PCR analysis

Aliquots of 2 mg of mRNA were reverse-transcribed using a PrimeScript RT reagent Kit with the gDNA Eraser Kit according to the manufacturer’s instructions (TaKaRa BioTechnology, China). SYBR Green-based qPCR was then performed on an ABI ViiA 7 Real-Time PCR system (Applied Biosystems, Foster City, CA, USA). *GAPDH* was used as the endogenous control, and all reactions were performed in triplicate. Relative gene expression was calculated using the comparative cycle threshold (2^−ΔΔCT^) method. PCR cycling conditions consisted of 5 min at 95°C followed by 40 cycles of 15 s of denaturation at 95°C, 30 s of annealing at 55°C, and 30 s of extension at 72°C.

## Results

### Cisplatin toxicity profile of A549 and A549/DDP cell lines

**Fig 1:**
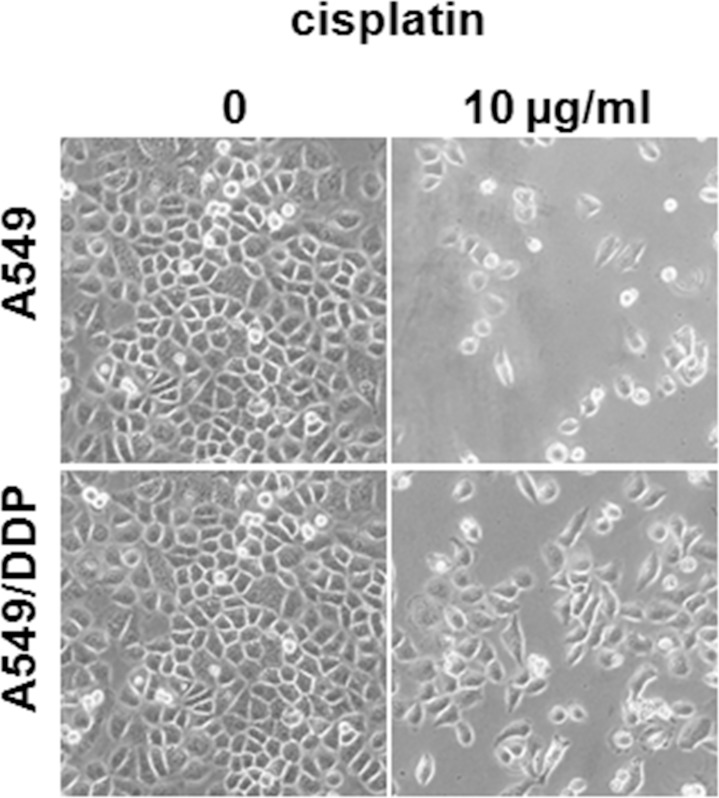
Cell morphology differences between A549 and A549/DDP cells. Micrographs showing A549 and A549/DDP cells cultured for 3 days in the absence or presence of cisplatin (10μg/mL).

**Fig 2:**
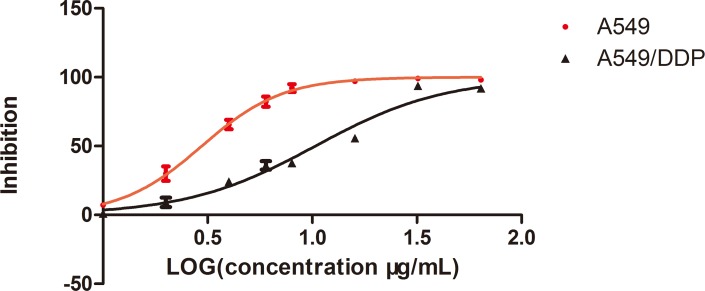
The inhibitory effects of different concentrations of cisplatin (DDP) on A549 and A549/DDP cells. Cell viability, as assessed by the CCK-8 assay, was determined 24 h after exposure of A549 or A549/DDP cells to increasing amounts of cisplatin. Results represent the average of triplicate wells and are representative of three independent experiments. Black bars and symbols, A549/DDP; red bars and symbols, A549.

To understand the underlying mechanism of cisplatin resistance, an *in vitro* cell model was established by repeated treatment of the adenocarcinomic human alveolar basal epithelial cell line A549 with a gradually increased cisplatin concentration. We designated the resistant cells as A549/DDP cells, and the cell morphology and growth rate did not differ significantly from the parent cell line under the inverted microscope (Olympus, Tokyo, Japan) (Fig 1). The single cell-derived clones for both the A549 and A549/DDP cell lines were obtained by a limiting dilution strategy and used for the subsequent experiments. The cisplatin toxicity profiles of the A549 and A549/DDP cell lines were assessed; the 50% inhibitory concentration value (mean ± SD) of cisplatin in A549 and A549/DDP cells was 2.965 ± 0.3μg/mL and 10.35 ± 0.35μg/mL, respectively, and the resistant index was 3.49 (*p* < 0.05). The dose–response growth inhibitory curve was plotted and is shown in Fig 2.

### Transcriptome sequencing analysis

**Fig 3:**
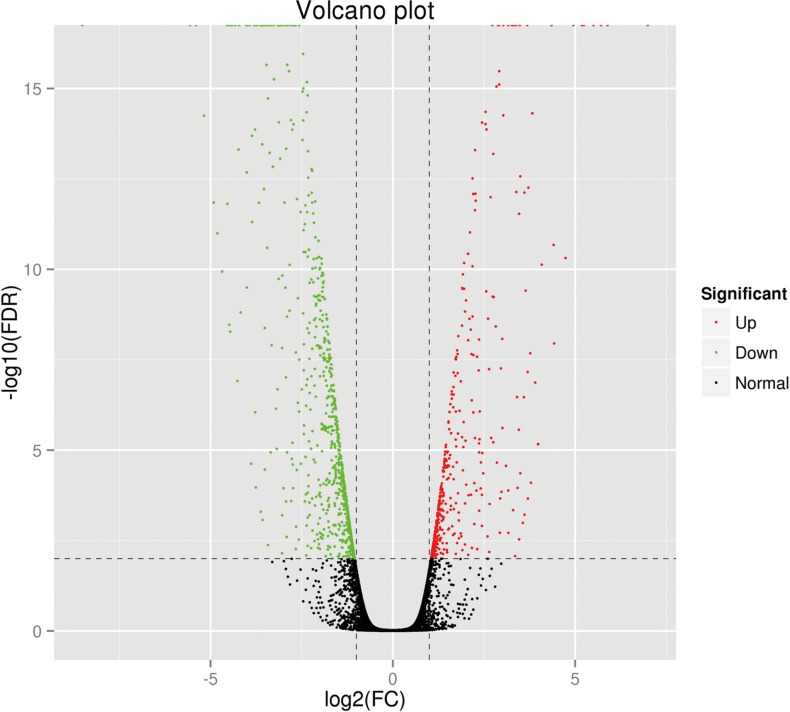
Distribution of the differentially expressed genes shown as a volcano plot. Each point represents a gene. The green dots represent the down-regulated differentially expressed genes, red dots represent the up-regulated differentially expressed genes, and black dots represent non-differentially expressed genes.

A total of 18.04 GB clean data were acquired after removing reads with adapters, unknown nucleotides, and low-quality reads. The Q30 percentage (sequencing error rate < 0.1%) was 95.77%. An overview of the sequencing and assembly statistics is shown in Table 1. The average sequencing depth was approximately 50 times the human transcriptome (approximately 113 million bp, based on the total length of the uniquely annotated exon region in the Ensembl database). TopHat software was used to align the reads to the reference human genome GRCh37. The proportion of reads that mapped to the reference genes was 87.07% for A549 cells and 86.02% for A549/DDP cells. The details of the mapping results are provided in Table 2. Transcript abundances were normalized using the RSEM package, and the fragments per kilobase of transcript per million fragments mapped value (FPKM) was estimated. On the basis of the selection criteria (q value < 0.005 and |log2 (fold change)| > 2), a total of 1214 DEGs were identified, 656 of which were upregulated and 558 were downregulated. The degree of expression change of these DEGs between the two samples is shown as a volcano plot in Fig 3.

**Table 1:** Statistical summary of transcriptome sequencing.

Samples	A549	A549/DDP
Raw reads	29,777,369	31,765,138
Clean reads	29,583,989	31,162,940
Clean bases	8,751,157,292	9,288,174,874
GC Content	56.96%	54.22%
%≥Q30	94.38%	93.78%

**Table 2:** Statistical summary of the mapping results.

Samples	A549	A549/DDP
Total Reads	53,211,580	50,236,010
Mapped Reads	46,330,867 (87.07%)	43,211,293 (86.02%)
Uniq Mapped Reads	42,089,964 (79.10%)	39,180,611 (77.99%)
Multiple Map Reads	4,240,903 (7.97%)	4,030,682 (8.02%)
Reads Map to '+'	23,033,497 (43.29%)	21,478,107 (42.75%)
Reads Map to '-'	23,067,281 (43.35%)	21,499,267 (42.80%)

### GO enrichment analysis

**Fig 4:**
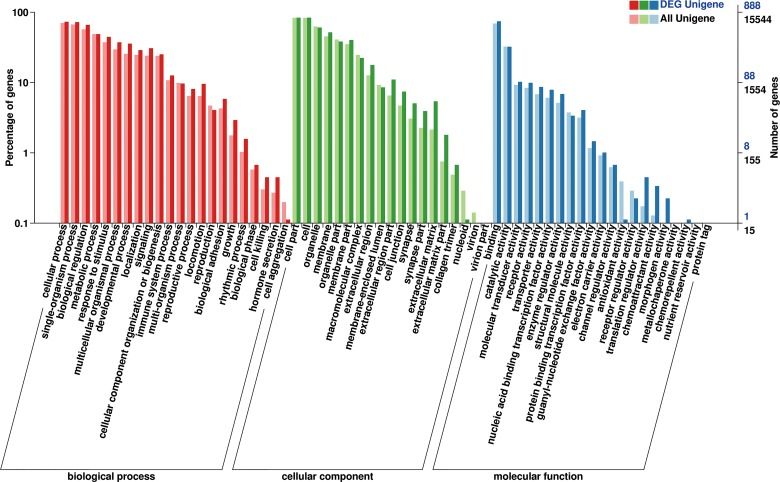
GO classification. Annotation statistics of differentially expressed genes in the secondary node of GO. The horizontal axis shows the secondary nodes of three categories in GO. The vertical axis displays the percentage of annotated genes versus the total gene number. The red columns display annotation information of the total genes and the blue columns represent annotation information of the differentially expressed genes only.

GO enrichment analysis of the DEGs was implemented by the GOseq R package based on the Wallenius non-central hyper-geometric distribution. A total of 1133 unigenes annotated in the GO database were distributed into three main functional biological categories: biological process, cellular component, and molecular function (Fig 4). In the cellular component category, 1071 DEGs (98.34% of all DEGs) were distributed in the cell or cellular part sub-category. In the molecular function category, the highest proportions of DEGs were distributed in the binding sub-categories (946 genes, representing 89.16% of all DEGs). In the biological processes category, the genes involved in cellular processes accounted for the greatest proportion (1063, representing 97.61% of all DEGs).

### COG enrichment analysis

**Fig 5:**
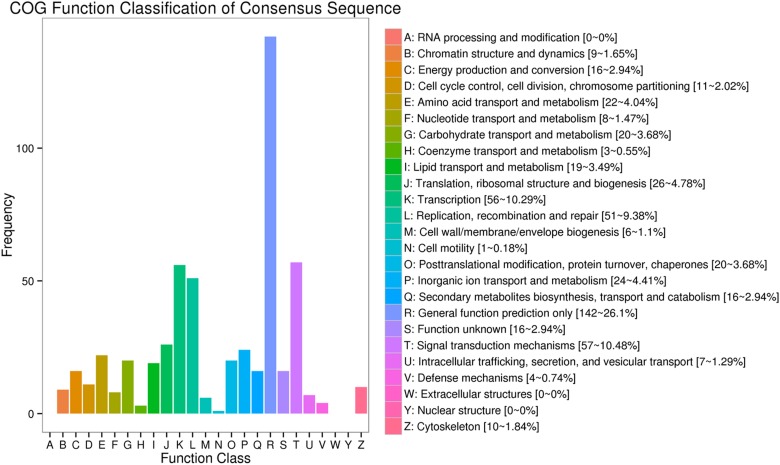
COG function classification of the consensus sequences. The COG categories are shown on the horizontal axis and gene numbers and proportions are plotted on the vertical axis.

COG is an orthologous gene product database wherein every protein is assumed to have evolved from an ancestral protein, and the whole database is built on coding proteins. Of all DEGs, 365 annotated unigenes were distributed into 26 COG functional classes (Fig 5). The top five DEGs-enriched categories were general function prediction only (142, representing 26.1% of all genes); signal transduction metabolism (57, representing 10.48% of all genes); transcription (56, representing 10.29% of all genes); replication, recombination, and repair (51, representing 9.38% of all genes), and translation, ribosomal structure, and biogenesis (26, representing 4.78% of all genes), suggesting that cell proliferation and replication were greatly affected in A549/DDP cells.

### KEGG pathway enrichment analysis

**Fig 6:**
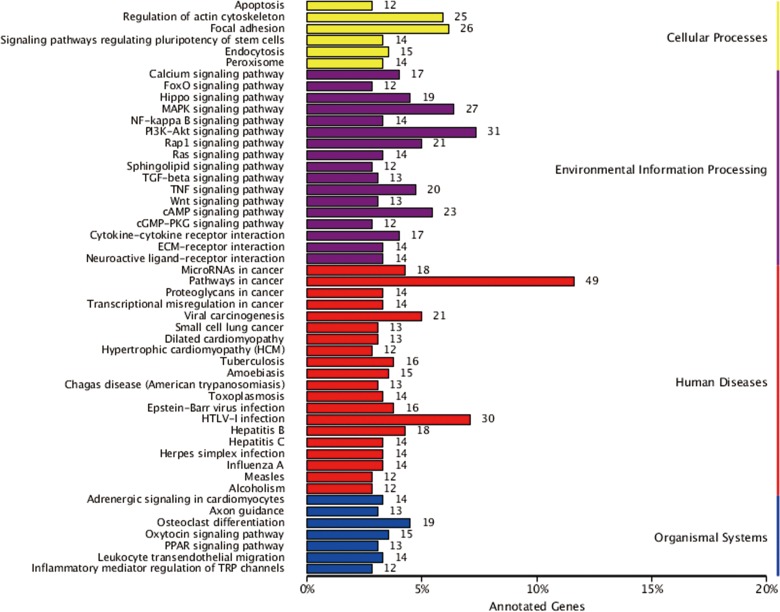
KEGG categories of differentially expressed genes. The vertical axis lists the names of the metabolic pathways in the KEGG database, and the horizontal axis shows the proportion of annotated genes in each pathway versus the total number of annotated genes.

**Fig 7:**
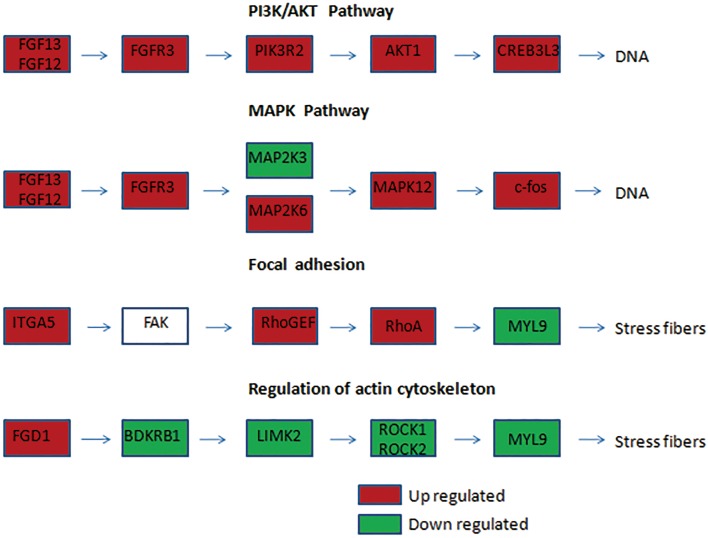
Significant deregulated key signaling nodes of most DEGs enriched pathways. Significant deregulated key signaling nodes of PI3K/AKT, MAPK, Focal Adhesion and Actin Cytoskeleton pathway.

**Fig 8:**
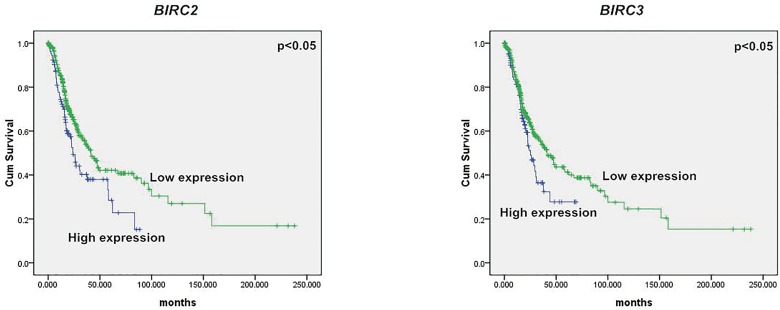
Correlation of BIRC2 or BIRC3 expression and DFS of patients with lung adenocarcinoma. Low expression of BIRC2 or BIRC3 is associated with improved DFS. Green and blue lines indicated low and high expression groups, respectively. *P* <0.05 was considered to be statistically significant.

KEGG is a database resource for understanding high-level functions and utilities of a biological system such as the cell, organism, and ecosystem from molecular-level information, especially large-scale molecular datasets generated by genome sequencing and other high-throughput experimental technologies (http://www.genome.jp/kegg/)[15]. In the present study, 741 DEGs were distributed into 260 pathways in the KEGG database. Fig 6 summarizes the top 50 DEGs-enriched pathways. Among them, the pathways in cancer included the largest number of DEGs. The other DEGs-enriched pathways included cell proliferation, signaling, and transduction pathways such as the PI3K-AKT, mitogen-activated protein kinase (MAPK), and cell invasion pathways, including those involved in regulation of the actin cytoskeleton and focal adhesion. Fig 7 summarizes the significantly deregulated key signaling nodes of these pathways. Furthermore, we downloaded the mRNA expression data from TCGA database and systematically evaluated the expression of genes of the DEGs enriched pathways and their correlation with patients’ survival in over 400 lung adenocarcinoma samples of the TCGA database. We found that low expression of BIRC2 or BIRC3 was associated with improved DFS which is in good accordance to our findings (Fig 8).

### qPCR verification

**Fig 9:**
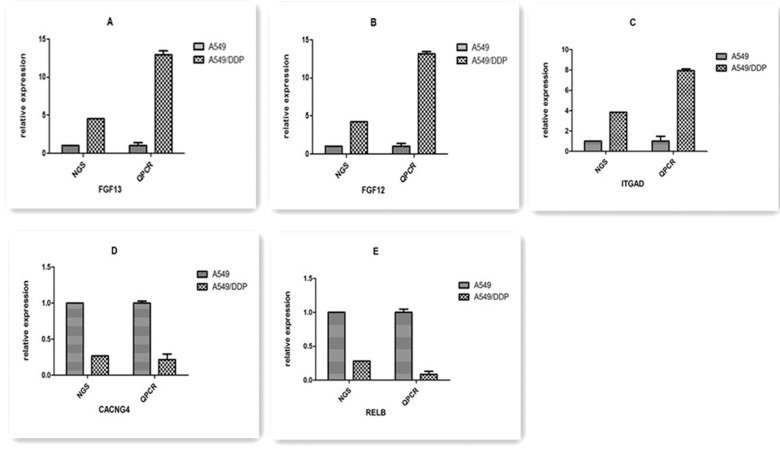
The differentially expressed genes detected by transcriptome sequencing confirmed by qRT-PCR. qRT-PCR was performed for 5 genes that were identified to be differentially expressed between A549 and A549/DDP cells. The expression level of each gene was normalized to the level in A549 cells. A–E: *FGF13*, *FGF12*, *ITGAD*, *CACNG4*, and *RELB*, respectively.

To experimentally confirm the results of transcriptome sequencing, we randomly selected five candidate genes (*FGF13*, *FGF12*, *ITGAD*, *CACNG4*, and *RELB*) among the top 5 DEGs-enriched KEGG pathways, and qRT-PCR was performed to evaluate their mRNA expression levels in A549/DDP cells and parental A549 cells. Highly concordant results were observed between transcriptome sequencing and qPCR, as shown in Fig 9.

### Validation in HepG2/DDP cell line

**Fig 10:**
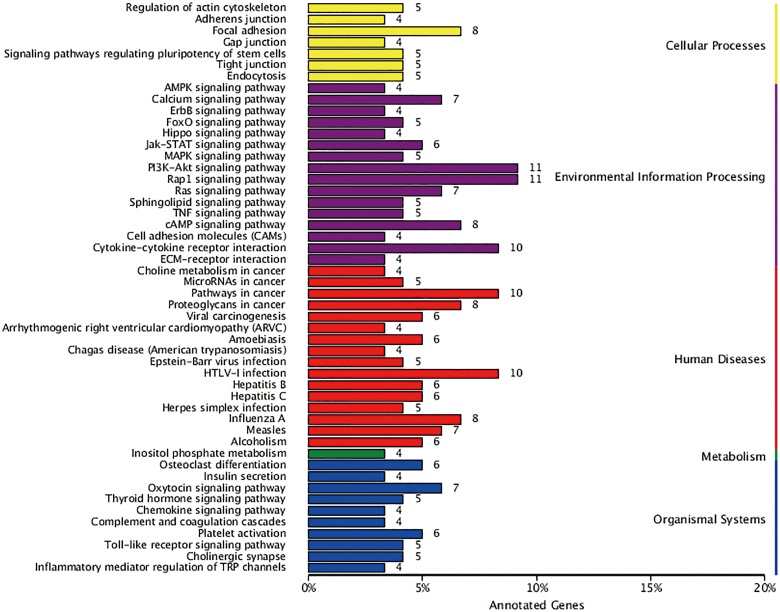
KEGG categories of HepG2/DDP cells DEGs. The vertical axis lists the names of the metabolic pathways in the KEGG database, and the horizontal axis shows the proportion of annotated genes in each pathway versus the total number of annotated genes.

To further validate our findings, whole-transcriptome sequencing analysis of hepatocellular carcinoma (HepG2) and HepG2/DDP cells was performed. The cisplatin-resistant HepG2/DDP cell line was previously established by the team of our lab from its parental cell line by step-dose selection *in vitro*. The transcriptome sequencing results showed that 372 genes were significantly differentially expressed between the cisplatin-resistant cell line and its parental cell line according to the selection criteria (q value < 0.005 and |log2 (fold change)| > 2), of which 129 were upregulated and 243 were downregulated. Among them, 219 annotated unigenes were distributed into 260 pathways in the KEGG database. Fig 10 summarizes the top 50 DEGs-enriched pathways. Among them, some DEGs enriched pathways were consistent with the results we mentioned above. Remarkably, PI3K-AKT and pathways in cancer were the top DEGs enriched pathways in both cell types. Besides, focal adhesion, mitogen-activated protein kinase (MAPK), and regulation of the actin cytoskeleton pathways also include a number of recurrent DEGs. The results together validated the findings acquired form A549/DDP cell lines.

## Discussion

In this study, we aimed to identify the genes associated with acquired resistance to cisplatin for the development of targeted therapy in the clinical treatment of lung cancer. First, we screened DEGs between the A549/DDP and A549 cell lines by transcriptome sequencing. Our results showed that 1214 genes were significantly differentially expressed between the cisplatin-resistant cell line and its parental cell line, 656 of which were upregulated and 558 were downregulated. Second, the function of the DEGs was annotated by reference to the COG, GO, and KEGG databases, and the top 5 DEGs-enriched pathways in the KEGG database were analyzed to elucidate the resistance mechanism. Finally, qRT-PCR was used to validate all of the RNA sequencing results.

We used an NGS technique to screen DEGs, which has proven to be a powerful tool for investigating the mechanisms of biological processes owing to its improved efficiency, cost benefits, and high-throughput data production[13]. The deep transcriptome sequencing of total RNA provides a comprehensive sequence resource for the conservation of genetic information and enrichment of the genetic database, which could also provide highly quantitative estimates for known individual gene species with potential for the discovery of novel genes, including those with a low frequency of occurrence [16]. Our screen is robust at identifying pathways associated with platinum resistance at a global level based on the whole-transcriptome sequencing analysis.

Annotation of the function of the DEGs in the KEGG database revealed that there are five pathways enriched with the most DEGs, including pathways in cancer, the PI3K-AKT pathway, MAPK pathway, and those involved in the regulation of the actin cytoskeleton and focal adhesion. We concluded that these five pathways may have a closer association with chemoresistance in lung cancer than other pathways, and we plan to focus on the DEGs in these pathways in future work to identify candidate genes for reversing cisplatin resistance.

Pathways in cancer represent a comprehensive network of the integration of all pathways related to tumorigenesis and cancer progression, which are systematically composed of a number of signaling pathways such as PI3K-AKT, MAPK, WNT, apoptosis, and others pathways associated with cancer cell proliferation, invasion, and metastasis. Significant DEGs (|log2 (fold change)| >4) in this pathway included *FGF13*, *FGF12*, *FGF18*, *MMP2*, and *MMP9*. *FGF13*, *FGF12*, and *FGF18* are members of the fibroblast growth factor (FGF) family, which are involved in a variety of biological processes, including cell proliferation, tumor growth, and invasion. A recent study demonstrated that FGF13 plays a pivotal role in mediating the resistance to platinum drugs in cervical cancer cells[17]. Matrix metalloproteinases (MMPs) are suggested to be correlated with the metastatic ability of cancer cells. In particular, abnormal expression of MMP2 and MMP9 has been frequently detected in solid tumor tissues and is associated with tumor metastasis in many cancer types[18].

PI3K/AKT is a crucial pathway regulating many biological activities such as cell proliferation and survival, motility and migration, and tumor cell invasion. The PI3K/AKT pathway has also been shown to be correlated with chemoresistance in multiple types of cancer. For example, ADAM17-induced chemoresistance drug resistance in hepatocellular carcinoma cells [19]and RLIP76-induced drug resistance in pancreatic cancer cells [20]were regulated by PI3K/AKT pathway. Numerous studies have confirmed that PI3K/AKT can confer resistance to DDP-based treatment in cervical cancer[21, 22], lung cancer[22], and ovarian cancer[23]. In our study, four genes involved in this pathway, including *NFKB1*, *PPP2R5B*, *PPP2R2B*, and *CCNE2*, were significantly deregulated in the cisplatin-resistant cell line. NFKB1 is a subunit of nuclear factor-kappa B (NFκB), an important regulator of genes controlling a variety of cell survival processes such as proliferation and apoptosis. Activation of the NFκB gene has been implicated in many human cancers[24]. Luo et al. showed that resistance to cisplatin treatment was partly due to the activation of cell survival genes such as NFκB and the subsequent loss of p53[25]. PPP2R5B and PPP2R2B belong to the protein phosphatase 2A family, which is implicated in the negative regulation of AKT protein, and consequently reduces cancer cell growth and division[26, 27]. CCNE2 belongs to the highly conserved cyclin family, whose members are characterized by a dramatic periodicity in protein abundance through the cell cycle. A previous study showed that CCNE2 promoted the proliferation, invasion, and migration of non-small cell lung cancer cells[28].

The MAPK signaling pathway is known to be a key regulator of cell proliferation and apoptosis. There are three major subfamilies of the MAPK family: the extracellular signal-regulated kinases, c-Jun N-terminal kinases, and p38 kinases. The importance of the activation of MAPKs in the cellular response to cisplatin and the development of cisplatin resistance has been gradually proven in recent years. Some researchers have even proposed that MAPK activation is a major component determining the cell fate in response to cisplatin[29, 30]. Wang et al. demonstrated that STC2 could regulate cell proliferation, apoptosis, and cisplatin resistance in cervical cancer by activating the MAPK signaling pathway[31]. Xie et al. found that treatment of the drug-resistant ovarian cancer cell line SKOV3/DDP with the p38 MAPK inhibitor SB203580 significantly improved the sensitivity to cisplatin. A recent study showed that the MAPK pathway is also closely associated with the chemoresistance of colorectal cancer therapy[32]. We found several DEGs that play important roles in the MAPK pathway, such as *MAPK12*, *FOS*, *MAP2K3*, and *MAP2K6*. *MAPK12* belongs to the p38 MAPK family, which is implicated in many cellular processes, including inflammation, differentiation, cell growth, cell cycle, and cell death[33]. *FOS* is a downstream gene in the MAPK pathway, which has a direct relationship with cancer cell proliferation and differentiation[34]. MAP2K6 and MAP2K3 are members of the MAPK kinase family, which phosphorylate and thus activate p38 MAPK[35].

Regulation of the actin cytoskeleton and focal adhesion pathways is essential for cell adhesion, cell motility, and morphological changes that can directly influence tumor invasion and metastasis. In addition, a cell adhesion-based mechanism could protect cells from various cytotoxic agents, including cisplatin and taxol[36]. Our transcriptome sequencing results identified several DEGs involved in cell adhesion, such as *BIRC2*, *BIRC3*, and *MLCK*, which could influence tumor invasion or metastasis. Previous studies of tumor metastasis suggested that strongly and weakly invasive cancer cells differ in terms of the reorganization of the actin cytoskeleton[37]. Chen et al. found that knockdown of the expression of the actin cytoskeleton protein ezrin contributed to sensitizing lung cancer cells to cisplatin and pirarubicin[38].

The focal adhesion pathway is critical for cell adhesion and maintenance of the tissue architecture. FAK is a cytoplasmic non-receptor tyrosine kinase, and its activation is accompanied by the accumulation of focal adhesion molecules[39]. FAK plays a pivotal role in integrin-mediated signal transduction, including the regulation of cell survival[40]. Activated FAK could bind multiple intracellular proteins to invoke several downstream signaling pathways such as the PI3K/AKT and MAPK pathways, thus stimulating tumor cell proliferation and inhibiting apoptosis, which is a major mechanism of tumor drug resistance[39]. In our study, *BIRC2*, *BIRC3*, and *MLCK* were found to be significantly deregulated. BIRC2 and BIRC3 are members of the IAP family of proteins that inhibit apoptosis by binding to the tumor necrosis factor receptor-associated factors TRAF1 and TRAF2. Moreover, BIRC3 has been reported as a novel inducer of platinum resistance in ovarian carcinoma cells[41]. The organization and stiffness of the cytoskeleton are determined in large part by the forces generated by actin and myosin II (MLC20), which are catalyzed by MLCK (myosin light chain kinase). Moreover, MLCK is responsible for the high proliferative and metastatic ability of breast cancer cells[42].

In conclusion, we have identified several pathways (PI3K/AKT, MAPK, regulation of actin cytoskeleton, focal adhesion) and DEGs (*MMP2*, *MMP9*, *FGF13*, *FGF12*, *NFKB1*, *PPP2R5B*, *PPP2R2B*, *CCNE2*, *MAPK12*, *FOS*, *MAP2K3*, *MAP2K6*, *BIRC3*, *BIRC2*, and *MLCK*) associated with cisplatin resistance in lung cancer cells. Our study provides new promising biomarkers for lung cancer prognosis and potential therapeutic targets for lung cancer treatment. Further studies are needed to validate the functions of the identified pathways and genes to obtain more confirmed biomarkers for predicting and overcoming the cisplatin resistance in the clinical treatment of lung cancer.
